# Pain, functional disability, and their Association in Juvenile Fibromyalgia Compared to other pediatric rheumatic diseases

**DOI:** 10.1186/s12969-019-0375-9

**Published:** 2019-11-06

**Authors:** Mark Connelly, Jenifer E. Weiss, for the CARRA Registry Investigators, L. Abramson, E. Anderson, M. Andrew, N. Battle, M. Becker, H. Benham, T. Beukelman, J. Birmingham, P. Blier, A. Brown, H. Brunner, A. Cabrera, D. Canter, D. Carlton, B. Caruso, L. Ceracchio, E. Chalom, J. Chang, P. Charpentier, K. Clark, J. Dean, F. Dedeoglu, B. Feldman, P. Ferguson, M. Fox, K. Francis, M. Gervasini, D. Goldsmith, G. Gorton, B. Gottlieb, T. Graham, T. Griffin, H. Grosbein, S. Guppy, H. Haftel, D. Helfrich, G. Higgins, A. Hillard, J. R. Hollister, J. Hsu, A. Hudgins, C. Hung, A. Huttenlocher, N. Ilowite, A. Imlay, L. Imundo, C. J. Inman, J. Jaqith, R. Jerath, L. Jung, P. Kahn, A. Kapedani, D. Kingsbury, K. Klein, M. Klein-Gitelman, A. Kunkel, S. Lapidus, S. Layburn, T. Lehman, C. Lindsley, M. MacgregorHannah, M. Malloy, C. Mawhorter, D. McCurdy, K. Mims, N. Moorthy, D. Morus, E. Muscal, M. Natter, J. Olson, K. O’Neil, K. Onel, M. Orlando, J. Palmquist, M. Phillips, L. Ponder, S. Prahalad, M. Punaro, D. Puplava, S. Quinn, A. Quintero, C. Rabinovich, A. Reed, C. Reed, S. Ringold, M. Riordan, S. Roberson, A. Robinson, J. Rossette, D. Rothman, D. Russo, N. Ruth, K. Schikler, A. Sestak, B. Shaham, Y. Sherman, M. Simmons, N. Singer, S. Spalding, H. Stapp, R. Syed, E. Thomas, K. Torok, D. Trejo, J. Tress, W. Upton, R. Vehe, E. von Scheven, L. Walters, J. Weiss, P. Weiss, N. Welnick, A. White, J. Woo, J. Wootton, A. Yalcindag, C. Zapp, L. Zemel, A. Zhu

**Affiliations:** 1 Children’s Mercy Kansas City, 2401 Gillham Road, Kansas City, MO 64108 USA; 2 Hackensack University Medical Center, 30 Prospect Avenue, WFAN, PC360, Hackensack, NJ 07601 USA; 3 CARRA, Inc., 555 East Wells Street, Suite 1100, Milwaukee, WI 53202-3823 USA

**Keywords:** Fibromyalgia, Pediatric rheumatic disease, Pain, Functional disability, Registry

## Abstract

**Background:**

Severe pain and impairments in functioning are commonly reported for youth with juvenile fibromyalgia. The prevalence and impact of pain in other diseases commonly managed in pediatric rheumatology comparatively have been rarely systematically studied. The objective of the current study was to determine the extent to which high levels of pain and functional limitations, and the strength of their association, are unique to youth with juvenile primary fibromyalgia syndrome/JPFS) relative to other pediatric rheumatic diseases.

**Methods:**

Using data from 7753 patients enrolled in the multinational Childhood Arthritis and Rheumatology Research Alliance (CARRA) Legacy Registry, we compared the levels and association of pain and functional limitations between youth with JPFS and those with other rheumatic diseases.

**Results:**

Pain levels were rated highest among youth with JPFS (M = 6.4/10, SD = 2.4) and lowest for juvenile dermatomyositis (M = 1.7/10, SD = 2.2), with pain significantly higher in the JPFS group than any other pediatric rheumatic disease (effect sizes = .22 to 1.05). Ratings on measures of functioning and well-being also were significantly worse for patients with JPFS than patients with any other rheumatic disease (effect sizes = .62 to 1.06). The magnitude of association between pain intensity and functional disability, however, generally was higher in other rheumatic diseases than in JPFS. Pain was most strongly associated with functional limitations in juvenile dermatomyositis, juvenile idiopathic arthritis, and mixed connective tissue disease.

**Conclusions:**

JPFS is unique among conditions seen in pediatric rheumatology with regard to ratings of pain and disability. However, pain appears to be comparably or more highly associated with level of functional impairment in other pediatric rheumatic diseases. Pain in childhood rheumatic disease thus would benefit from increased prioritization for research and treatment.

## Background

Juvenile primary fibromyalgia syndrome **(**JPFS) affects up to 6% of otherwise healthy children and commonly leads to subspecialty evaluation with a pediatric rheumatology provider [1–3]. The primary symptom of JPFS is widespread musculoskeletal pain that persists in the absence of an underlying inflammatory disease or other medical condition. In at least a subset of youth with JPFS, pain is associated with significant and enduring impairment in the ability to participate in usual activities and maintain positive well-being [4–7]. Reducing this functional impairment often is regarded as a primary goal of treatment for JPFS. This treatment goal is based in part on the clinical tenet that the perception of pain often improves once functional ability is optimized [8]. Thus, a strong association between severity of functional disability and pain in JPFS is assumed in treatment and has been partly borne out in extant research.

For other medical conditions commonly managed in pediatric rheumatology, levels of impairment in functioning and well-being also can be substantial. For example, children with juvenile dermatomyositis (JDM), systemic lupus erythematosus (SLE), or juvenile idiopathic arthritis (JIA) have significantly reduced scores on measures of health-related quality of life (HRQOL) relative to healthy peers [9–12]. The prevalence and severity of pain in most pediatric rheumatic diseases, however, rarely has been systematically studied [13]. Consequently, the extent to which pain is associated with indices of functioning across pediatric rheumatic diseases is largely unknown. Determining this would help inform the conceptualization and comprehensive treatment of JPFS and other pediatric rheumatic diseases.

Multi-site patient registries offer an optimal means of comparing clinical data across conditions. Registries use observational study methods to collect high quality data in naturalistic clinical settings [14]. Data elements typically are specified consistently, captured uniformly across sites, and are considered to accurately represent the clinical status of patients. For diseases with relatively low prevalence in particular, multi-site registries can be instrumental in ensuring sufficient sample sizes to make generalizable inferences from comparative observational research.

For the current study, we used data from the Childhood Arthritis and Rheumatology Research Alliance (CARRA) Legacy Registry to achieve the following aims: (a) to quantify the occurrence and intensity of pain across a representative sample of pediatric patients diagnosed either with a chronic idiopathic musculoskeletal pain condition (JPFS) or another rheumatic disease; and (b) to determine if the strength of relationship between pain and functioning is unique to JPFS or comparable across diseases seen in pediatric rheumatology. We hypothesized that levels of pain and disability, and the magnitude of their association, would be uniquely high among youth with JPFS relative to youth with other rheumatic diseases.

## Patients and methods

### Study design

The study was an observational study comprising a retrospective review of data. Data were originally obtained as part of the CARRA Legacy Registry for patients who enrolled and had an initial assessment visit between May 2010 and May 2014 (the time period of active registry recruitment for the CARRA Legacy Registry). The CARRA Legacy Registry is the largest multi-site clinical database of children and adolescents with a rheumatic disease. Data from this registry thus are regarded to be generalizable and ideal for comparing disease characteristics and/or outcomes across diagnoses.

### Participants and sites

Patients from 56 pediatric rheumatology centers in North America contributed data to the CARRA Legacy Registry. Conditions represented in this registry and included for the current study comprised Juvenile Primary Fibromyalgia Syndrome (JPFS), mixed connective tissue disease (MCTD), SLE, JDM, JIA, and central nervous system vasculitis. To be eligible for the registry, patients had to be under the age of 18 years at the time of symptom onset and under the age of 21 years at the time of giving consent to participate. For the current study, we included children and adolescents who were between the ages of 5–21 (inclusive) at the time of consent. For JPFS, patients needed to meet American College of Rheumatology (ACR) criteria (if age 18 and above) or Yunus and Masi criteria [15–17]. Established diagnostic criteria were applied for each of the other pediatric rheumatic diseases.

### Procedure

Recruitment of patients to participate in the registry was conducted by site research coordinators during new or follow-up outpatient rheumatology visits. Written informed consent and assent (for those above 9 years old) was obtained for all patients agreeing to enroll in the registry. Standardized case report forms were used for data collection at each site. Data sources included medical chart review, physician assessment, and physician/parent/patient completion of subjective measures. Data recorded on the case report forms were manually entered into local databases by site coordinators. Entered data were integrated into a centralized i2b2 data warehouse from which approved registry investigators could request deidentified databases for study analyses [18]. Procedures for the CARRA Legacy Registry were approved by the Institutional Review Board at each participating site. The current study was approved by the Institutional Review Board at Hackensack University Medical Center.

### Measures

#### Patient characteristics

Information on patient- or parent-reported demographics (age, sex, race, ethnicity, and family household income), diagnosis, date of symptom onset, date first seen by a pediatric rheumatologist, body mass index, and family history of fibromyalgia and autoimmune disease was recorded on standardized case report forms at the index visit.

#### Pain intensity

Patients ages 10 and above were asked to rate the average intensity of pain associated with his/her condition over the past week using a 0–10 numeric rating scale, with 0 being “no pain” and 10 being “very severe pain.” The numeric rating scale is considered a well-established measure for measuring the presence and intensity of pain in pediatric populations [19]. For children ages 5–9, the Faces Pain Scale Revised was used to quantify self-reported pain intensity [20].

#### Measures of functioning

The Childhood Health Assessment Questionnaire was completed by patients or caregiver proxies (for children younger than 10) as a measure of impact of disease on daily functioning over the past week [21]. This questionnaire has eight categories: dressing and grooming, arising, eating, walking, hygiene, reach and grip. Item scores range from 0 to 3, with higher scores indicating worse functioning. Patients (or caregiver proxies for children under 10) also completed two global ratings pertaining to the patient’s general functioning and well-being [22]. On the first, patients/caregivers were asked to rate the patient’s overall health on a 5-point scale (“excellent” to “very poor”), with higher scores indicating poorer health-related quality of life. On the second, patients were asked to consider all the ways their condition affects them and then use a 0–10 scale (“very well” to “very poor”) to rate how they are currently doing; higher numbers indicate poorer overall well-being. Clinicians also rated functional ability using the 4-point ACR functional class rating (1 = “able to perform usual activities of daily living; 4 = “limited in ability to perform usual self-care, vocational, and avocational activities”) [23].

#### Disease activity

The physician global assessment of disease activity (MDGA) was used to quantify disease activity on a 0–10 numeric rating scale, with “0” representing “inactive disease” and “10” representing “very active disease.” [24]

### Analyses

Descriptive statistics (frequency counts and percentages, means or medians, and standard deviations) were used to summarize data from index visits on patient characteristics, pain intensity, and indices of functional disability across all conditions. Differences in baseline patient characteristics as a function of group (JPFS versus other rheumatic diseases combined) were evaluated using correlations, chi-square analyses, or independent samples t-tests as applicable. Omnibus general linear models were used to compare groups (JPFS versus other rheumatic diseases combined) on pain severity and on each of the functioning measures. In these models, age and MDGA were specified as covariates. If the overall test was significant, the JPFS group was compared to each of the individual rheumatic diseases included in the combined group. Effect sizes for group differences are reported based on Cohen’s *d* formula. Fisher’s r-to-z transformation was used to compare disease groups on the correlation between pain and functional measures. SPSS® software (IBM) version 24.0 was used for all analyses.

## Results

### Patient characteristics

Data on patient characteristics for the cohort are presented in Table 1. Across all conditions there were 7753 patients, primarily female, with ages ranging from 5 to 21 years inclusive (M = 13.0, SD = 4.0). Of these, 201 were diagnosed with JPFS and 7552 were diagnosed with another rheumatic disease (Table 1). The patients with JPFS on average were significantly older than other patients with rheumatic diseases at the time of the index visit (M = 15.5 versus 13.0), at the time of reported symptom onset (M = 12.1 versus 8.3), and at the time of the first visit to a rheumatologist (M = 13.7 versus 9.2). A significantly higher proportion of females were represented among patients with JPFS (84%) than in other rheumatic diseases (72%). A significantly higher proportion of patients with a reported family history of fibromyalgia or autoimmune disease were represented among patients with JPFS (18 and 45%, respectively) than in other rheumatic disease (4 and 31%, respectively). Patients with JPFS on average had significantly greater body mass index (M = 24.2 versus 21.2). There were no other significant differences in measured patient characteristics between the JPFS cohort and other rheumatic disease cohort.

**Table 1:** Demographic data for the juvenile primary fibromyalgia syndrome cohort and other rheumatic disease cohort

Variable	Juvenile Primary Fibromyalgia Syndrome Cohort *(n* = 201)	Combined Rheumatic Disease Cohort (*n* = 7552)
Age (years)*	M = 15.5, SD = 2.2 (range 9–20)	M = 13.0, SD = 4.0 (range 5–21)
Sex*	84% female	72% female
Race	85% White	80% White
	8% Black/AA	10% Black/AA
	2% Asian 1% American Indian or	3% Asian 1% American Indian or
	Alaskan Native	Alaskan Native
	4% Other	6% Other
Ethnicity	17% Hispanic	13% Hispanic
Median family income interval	$75–$100,000	$50–$75,000
Age at symptom onset (years)*	M = 12.1, SD = 3.1 (range 2–18)	M = 8.3, SD = 4.7 (range 0.2–21)
Age at first rheumatologist visit (years)*	M = 13.7, SD = 2.8 (range 3–20)	M = 9.2, SD = 4.7 (range 0.2–21)
Body mass index*	M = 24.1, SD = 6.0 (range 13.7–50.7)	M = 21.2, SD = 5.8 (range 11.1–53.0)
Family history of fibromyalgia*	18% yes	4% yes
Family history of autoimmune disease*	45% yes	31% yes

*Significant group difference in mean or proportion, *p* < .05

### Comparison of pain across conditions

Table 2 provides average pain ratings as a function of each of the individual conditions included in the study. Nearly all (97%) of youth with JPFS reported current pain. The majority (69%) of patients having a condition other than JPFS also reported current pain at the index visit, but this proportion was significantly lower than that of the JPFS group, χ^2^(1, 7753) = 72.15, *p* < .01. Reported intensity of pain was significantly higher on average for patients with JPFS than for patients with other rheumatic diseases (M = 6.4, SD = 2.4 versus M = 2.6, SD = 2.7; *F* (1, 7749) = 167.9, p < .01. effect size = 1.49). Regardless of diagnosis, the mean pain intensity scores reported at the index visit spanned the entire range from 0 to 10. All conditions also had at least some patients rating pain in the severe range (> 7/10). The proportion of patients reporting severe pain was significantly greater for those in the JPFS cohort relative to the other rheumatic diseases group (54.7% versus 11.7%), χ^2^(1, 7753) = 324.6, *p* < .01. Lowest pain ratings were observed for the group of patients diagnosed with juvenile dermatomyositis (M = 1.7/10, SD = 2.2).

**Table 2:** Means and standard deviations for pain and functioning measures by condition

Condition	Pain		HRQOL		Well-Being		ACR Functional Class		CHAQ	
	M	SD	M	SD	M	SD	M	SD	M	SD
JPFS (*n* = 201)	6.36	2.43	3.00	.83	5.08	2.19	1.60	.80	.76	.56
JIA (*n* = 5681)	2.69	2.66	2.15	.83	2.45	2.35	1.25	.54	.38	.54
SLE (*n* = 987)	2.40	2.61	2.40	.83	2.70	2.47	1.24	.57	.26	.46
JDM (*n* = 558)	1.66	2.21	2.21	.88	2.34	2.45	1.34	.76	.41	.67
Vasculitis (*n* = 187)	1.86	2.52	2.35	.89	2.56	2.42	1.27	.67	.27	.61
MCTD (*n =* 139)	2.96	2.35	2.37	.74	2.83	2.39	1.30	.56	.35	.49

Note: Values shown are unadjusted means and standard deviations. Higher pain scores reflect more severe pain. Higher scores on other measures indicate more severe impairments in functioning. JPFS = juvenile primary fibromyalgia syndrome; JIA = juvenile idiopathic arthritis; SLE = systemic lupus erythematosus; JDM = juvenile dermatomyositis; MCTD = mixed connective tissue disease

### Comparison of functional disability across conditions

Table 2 also provides summary data on the functioning measures by each of the individual conditions included in the study. After adjusting for physician-rated disease activity and age, ratings on measures of functioning were significantly worse for patients with JPFS than for patients with all other rheumatic diseases combined (effect sizes = .62 to 1.06). Specifically, patients with JPFS on average had significantly worse adjusted scores on the perceived well-being measure (M = 5.0 vs. 2.5, *F* (1, 7610) = 66.79, *p* < .01, effect size = .87), the CHAQ (M = .76 vs. .36, *F* (1, 7749) = 18.68, *p* < .01. effect size = .72), ACR functional class index (M = 1.6 versus M = 1.2, *F* (1, 7318) = 49.68, *p* < .01, effect size = .58), and the HRQOL measure (M = 3.0 versus M = 2.2, *F* (1, 7595) = 50.90, *p* < .01, effect size = .92).

### Relationship between pain and function

Table 3 displays the correlations between the measures of pain and functioning by each individual condition. In general, the relationship between level of pain and indicators of functional disability was significantly greater for children with other rheumatic diseases relative to patients with JPFS. The relationship of CHAQ functional disability scores to pain level was highest for patients with MCTD, SLE, JDM, and JIA patients (*r*s = .44–.59). The relationship between pain and ACR functional class scores was significantly stronger for patients with JDM and JIA (*r* = .44 and *r* = .36) than for those with JPFS (*r* = .24). For HRQOL, scores were significantly more strongly related to pain level for youth with JIA and MCTD (*r* = .51 and *r* = .55) compared to youth with JPFS (*r* = .40). Finally, relative to youth with JPFS, there was a significantly stronger relationship observed between pain and well-being ratings for JIA (*r* = .62 vs. *r* = .71, respectively).

**Table 3:** Correlations between measures of pain and functioning by condition

Condition	HRQOL	Well-Being	ACR Functional Class	CHAQ
JPFS (*n* = 201)	.40^*^	.62^*^	.24^*^	.34^*^
JIA (*n* = 5681)	.51^*†^	.71^*†^	.36^*†^	.54^*†^
SLE (*n* = 987)	.46^*^	.62^*^	.29^*^	.44^*†^
JDM (*n* = 558)	.48^*^	.57^*^	.44^*†^	.59^*†^
Vasculitis (*n* = 187)	.47^*^	.52^*^	.16^*^	.31^*^
MCTD (*n =* 139)	.55^*†^	.67^*^	.33^*^	.56^*†^

* *p* < .05 for correlation coefficient; ^†^ = correlation coefficient is significantly different (*p* < .05) from the JPFS group based on Fisher r-to-z transformation analysis. *JPFS* juvenile primary fibromyalgia syndrome, *JIA* juvenile idiopathic arthritis, *SLE* systemic lupus erythematosus, *JDM* juvenile dermatomyositis, *MCTD* mixed connective tissue disease

## Discussion

Patients with JPFS were found in this study to reliably report both higher levels of pain and more impairment in functioning than children with any other rheumatic disease. The degree to which pain is associated with impairment in functioning and well-being, however, was found to be variable across rheumatic conditions. Results suggested in particular that the occurrence and level of pain in patients with JIA, JDM, SLE, and MCTD is robustly linked to indicators of greater impairments in functioning.

This study further confirms that severe pain and impairment in functioning is typical in youth with JPFS who are seen in pediatric rheumatology clinics. This is a common observation in research on youth with idiopathic chronic musculoskeletal pain [4, 6, 25, 26]. A distinguishing feature of the current study, however, is that uniformly collected data were aggregated into a registry from multiple sites across North America. Thus, results are likely generalizable beyond the idiosyncrasies of a particular region or practice. Further, we were able to compare identical measures of pain and functioning across conditions managed in pediatric rheumatology. To our knowledge, such comparison has not been previously done. Results of these analyses suggest that levels of pain and functional impairment on average are more severe in JPFS than other conditions encountered in pediatric rheumatology. Similar findings were reported in a recent adult study comparing indices of HRQOL across rheumatic diseases; fibromyalgia was second only to rheumatoid arthritis in its impact on functioning [27]. There are multiple potential reasons for these findings. Youth with JPFS often are likely to have comorbid symptoms of anxiety and depression, which can further augment pain perception and problems with functioning and quality of life [6]. Pain in JPFS also is partly a function of “amplification” (or disordered regulation) of nociceptive input and is the primary symptom affecting youth with JPFS; this is not necessarily the case in other pediatric rheumatic diseases. Pain and impairments in functioning in JPFS may also be difficult to significantly modify with the resources available to most pediatric rheumatologists. Alternatively, children with a rheumatic disease may be more likely than youth with JPFS to downplay or underreport problems with pain and functioning to their care team out of concern that reporting such problems could lead to increased bothersome medical treatment.

The ability to compare the relationship between pain and indicators of functioning in the current study facilitated some unique findings. Pain in JPFS has a relatively well-established moderate association with functional limitations [4]. Patients with inflammatory rheumatic disease and connective tissue disorders (JIA, JDM, SLE, and MCTD), however, were observed in our study to have a comparatively stronger relationship between pain and functional disability. Identifying similarities in the manifestation of the disease process in these particular rheumatic diseases may help explain this finding. For example, it may be that the quality and/or location of pain experienced with these conditions is especially limiting for participating in the physical activities typically enjoyed by children and adolescents. Pain in these conditions, when high or enduring, may also result in aggressive disease-modifying treatments that have adverse consequences for functioning. Alternatively, pain may work synergistically with other disease symptoms (e.g., muscle weakness, arthritis) common in these conditions to further augment existing functional impairment. In the absence of further research, possible explanations remain speculative. Nevertheless, the results further establish the unique importance for assessing and treating pain in pediatric rheumatology. Managing disease activity understandably is the central focus in treating rheumatic disease [28]. Regardless of disease activity level, however, we observed a relatively robust relationship between pain and functional disability across most pediatric rheumatic diseases.

Use of data from the CARRA Legacy Registry enabled an evaluation of clinically relevant research questions that otherwise would have been difficult and costly to conduct. This registry provided the infrastructure for secure collection, storage, and sharing of uniformly collected data from multiple informants for over 9000 pediatric rheumatology patients [29]. Cross-condition comparisons were made possible by having included common data elements in the registry, supplemented by data collection forms specific to each disease. Despite these advantages, there also were limitations associated with use of registry data for the current study. Measures used to evaluate pain and functioning sacrificed comprehensiveness for efficiency and universality. The selected measures (e.g., the CHAQ) unlikely fully captures the nuances of pain and functional impairment for all conditions evaluated in the study. Patients enrolled in the registry also were a “convenience sample” being treated in pediatric rheumatology and were able to speak and read English. Further, not all 56 clinical sites contributed data on each condition in the registry; for example, fewer than half of sites contributed data on patients with JPFS. Patients in the fibromyalgia sample also were required to meet classification criteria commonly used in research (American College of Rheumatology criteria if > = 18 years old or Yunus and Masi diagnostic criteria if < 18 years). Thus, although use of the registry facilitated access to data from representative cohorts of patients, there may still be limits on the generalizability of findings. Data for the current study also were collected at a single occasion during any point of a patient’s disease course. Levels and associations of the variables studied may have varied had we evaluated them prospectively.

## Conclusions

Results of the current study further confirm that pain and impairment in indicators of functioning often are severe in youth with JPFS but may be even more connected with one another in patients with other rheumatic diseases including JIA, JDM, SLE, and MCTD. Pain appears to reliably relate to perceived impairments in typical daily functioning and overall well-being across these conditions. Historically pain has been less routinely assessed or targeted for treatment in many pediatric rheumatic disease relative to more objective indicators of disease activity. Pain and optimal approaches to minimize its impact also have been rarely studied in several of the pediatric rheumatic diseases. However, more routine incorporation of pain and other patient-reported outcomes into patient registries in part is helping to address this [29–31]. Our findings emphasize that pain across conditions commonly managed in pediatric rheumatology would benefit from continued increased prioritization in assessment, treatment and research.

## Data Availability

The data that support the findings of this study are available from the Childhood Arthritis and Rheumatology Research Alliance (CARRA, Inc.), but restrictions apply to the availability of these data and so are not publicly available. Data are available upon reasonable request from and with permission of CARRA, Inc. Policies pertaining to data requests and sharing for CARRA, Inc. are available here at the following link: https://carragroup.org/UserFiles/file/CARRA-DATA-SAMPLE-SHARING-POLICY-04November2016.pdf

## Abbreviations

ACR: American College of Rheumatology
CARRA: Childhood Arthritis and Rheumatology Research Alliance
HRQOL: health-related quality of life
JDM: Juvenile Dermatomyositis
JIA: Juvenile Idiopathic Arthritis
JPFS: Juvenile Primary Fibromyalgia Syndrome
MCTD: Mixed Connective Tissue Disease
MDAGA: physician global assessment of disease activity
SLE: Systemic Lupus Erythematosus
