# Correction to: Pain, functional disability, and their Association in Juvenile Fibromyalgia Compared to other pediatric rheumatic diseases

**DOI:** 10.1186/s12969-019-0397-3

**Published:** 2020-02-12

**Authors:** Mark Connelly, Jennifer E. Weiss, for the CARRA Registry Investigators

**Affiliations:** 1 Children’s Mercy Kansas City, 2401 Gillham Road, Kansas City, MO 64108 USA; 2 Hackensack University Medical Center, 30 Prospect Avenue, WFAN, PC360, Hackensack, NJ 07601 USA; 3 CARRA, Inc, 555 East Wells Street, Suite 1100, Milwaukee, WI 53202-3823 USA

**Correction to: Pediatr Rheumatol (2019) 17:72**

**https://doi.org/10.1186/s12969-019-0375-9**

Following publication of the original article [1], we have been notified that the corresponding author’s given name is spelled incorrectly. The given name, thus, should be as follows:

Jennifer E. Weiss.

The original article has been corrected
